# Fibrolipomatous hamartroma with macrodactyly in a 4 years old female patient: A case report

**DOI:** 10.1016/j.ijscr.2024.109680

**Published:** 2024-04-24

**Authors:** Hardisiswo Soedjana, Betha Egih Riestiano, Lisa Y. Hasibuan, Valdi Muharam Kusumadiningrat

**Affiliations:** Division of Plastic Reconstructive and Aesthetic Surgery, Department of Surgery, Faculty of Medicine, Universitas Padjadjaran, Bandung, Indonesia

**Keywords:** Case report, Fibrolipomatous hamartoma, Macrodactyly, Neural fibrolipoma

## Abstract

**Introduction:**

Neural fibrolipoma, also known as fibrolipomatous hamartoma (FLH), is a rare benign tumor that usually affects the upper limb and tends to develop near the n. median. FLH is a rare birth defect defined by the noncancerous growth of fibroadipose tissue around nerve bundles. These conditions are associated with abnormal bone growth, resulting in macrodactyly in about one-third of cases. The illness is medically referred to as macrodystrophia lipomatosa (MDL).

**Case presentation:**

A-4 years old girl presents with the index finger and thumb larger than the surrounding fingers and has been present since birth. Over time, the index finger and thumb continue to enlarge. This enlargement is accompanied by pain, a tingling sensation, and occasional bluish discoloration, especially at night. The patient is the third child out of four siblings, with a history of normal birth and no abnormalities in other parts of the body. The patient can grip objects in daily activities, but there is noticeable stiffness in the right hand.

**Discussion:**

FLH with macrodactyly is an uncommon abnormality that can manifest as either a minor lesion or a big mass affecting the entire extremity. Clinically, it is challenging to challenging to diagnose FLH in patient with macrodactyly.

**Conclusion:**

It is crucial to possess a thorough understanding of the distinctive histology and radiological findings in order to accurately diagnose and treat the condition.

## Highlights

• Neural fibrolipoma with macrodactyly is a rare condition
• Diagnosing neural fibrolipoma in patients with macrodactyly is tough
• Key histology and imaging insights are essential for accurate diagnosis and treatment

## 1 Introduction

Neural fibrolipoma, also known as fibrolipomatous hamartoma (FLH), is a rare benign tumor that usually develops in the upper limb and tends to arise around the median nerve. This disorder also affects the ulnar, radial, and brachial plexus and advance over a period of time.

Clinically, it appears as a flexible, progressively enlarging, spindle-shaped mass. The illness may be accompanied by pain, inflammation, and decreased sensitivity [1]. It is also known as neural fibrolipoma (NFL) as uncommon genetic disorder defined by the mild growth of fibroadipose tissue surrounding neural fibers. FLH develops from the abnormal growth of fatty tissue within the protective covering of a neuron. The predominant occurrence was an impingement of the median nerve, frequently caused by the gradual expansion of a tumor on the palmar surface of the wrist. This tumor exhibits characteristics of compressive neuropathy. This tumor possesses the ability to impact various nerves, including the n. ulnar, n. radial, and brachial plexus, but to a lower degree. FLH is linked to macrodactyly in around 27–67 % of cases. This disorder mainly affects the 2nd and 3rd fingers of the hand or foot, however it may infrequently involve numerous fingers. There have been a few documented instances of neural fibrolipoma occurring in the craniofacial region.

FLH is a rare, slowly growing, tumourlike condition that has been most commonly reported as involving the median nerve and its branches. Involvement of the ulnar nerve is rare and only a few cases have been reported. This lesion was described with different names such as “neurolipomatosis”, “neural fibrolipoma” and “macrodystrophia lipomatosa”. FLH may be seen associated with or without macrodactyly in the hand. Bibbo and Warren reported that upper extremity cases would typically present before the age of 25, usually without macrodactyly and without a documented history of trauma. Sex distribution including female-to-male ratio was reported equal and 2:1 for the FLH without macrodactyly, and with macrodactyly, respectively. FLH usually occur within the first two or three decades of life and the etiology is not clear. It is currently believed that FLH is, quite simply, a hamartoma because fibrous and fatty tissues are normal constituents of the connective tissue stroma of the nerve. These diseases are associated with abnormal bone formation, which leads to macrodactyly in around 33 % of instances. The medical term for this illness is macrodystrophia lipomatosa (MDL) [2,3].

Macrodactyly, also referred to as MDL, is a congenital disease that is not hereditary. It is defined by the excessive growth of fibroadipose tissue and often accompanied by the development of new bone in the periosteal and endosteal areas. The median nerve is frequently impacted, whereas the medical literature also documents the participation of other peripheral nerves. The histological examination reveals the existence of fully developed fatty and connective tissue infiltrating the epineural and perineural regions. Impinged neurons can display distinct pseudo-onion bulb formations and the production of metaplastic osseous tissue [2,3].

Fingeral macrodactyly is a rare congenital abnormality that appears at birth and gradually worsens as the skeleton develops normally. This extraordinary anomaly has three variations. The three forms of fingeral gigantism are as follows: type I, distinguished by the presence of lipofibromatous hamartoma in a peripheral nerve; type II, linked to neurofibromatosis; and type III, defined by hyperostotic characteristics. Arthrodesis of the interphalangeal joints may be required in adults, either through bone shortening or partial ray removal [4,5].

In instances of idiopathic or real macrodactyly, the finger's structures. All the components of the hand, such as the phalanges, neurons, arteries, and tendons, are expanded in a consistent manner as a result of the invasion of fibrofatty tissue. Neural territory-oriented macrodactyly is a specific type of macrodactyly characterized by the enlargement of the peripheral nerve that supplies the impacted area. These findings indicate that the illness may be attributable to a neurological origin [6].

This report will present the case of FLH oma associated with macrodactyly in a first decade female paient which was quite rare according to epidemiology and prevalence number mentioned before in previous research. Additionally, the patient was also provided with informed consent regarding potential publication and this work has been reported in line with the SCARE criteria [7].

## 2 Case presentation

In our detailed case presentation of the 4-year-old female patient with FLH, we observed and documented specific clinical signs and symptoms that warranted our attention. Upon examination, the patient exhibited pronounced enlargement of the index finger and thumb of the right hand, a hallmark sign of macrodactyly associated with FLH. This enlargement was not uniform; the thumb and index finger were disproportionately larger compared to the other fingers, with noticeable swelling and a firm texture upon palpation, indicating the presence of fibroadipose tissue.

The patient reported pain, which was more pronounced at night, and a tingling sensation along with occasional episodes of bluish discoloration of the affected fingers, suggesting compromised vascular supply or nerve compression. These symptoms were particularly evident in the anatomical areas surrounding the median nerve's path through the wrist and hand, which is commonly affected in FLH. The anatomical distribution of symptoms, coupled with the physical manifestations in specific fingers, provided crucial insights into the condition's localized impact on the hand's structure and function.

**Fig. 1:**
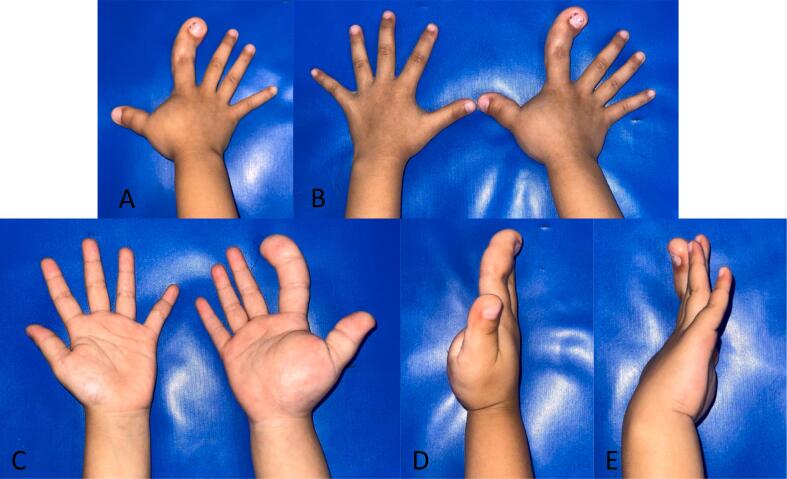
The macrodactylous right index finger and thumb. A. View of the dorsum of the right hand and affected index finger and thumb. B. View of the dorsum of the right and left hand. C. View of the palmar of the right and left hand. D. View of lateral thumb right hand. E. View of lateral small finger right hand.

**Fig. 2:**
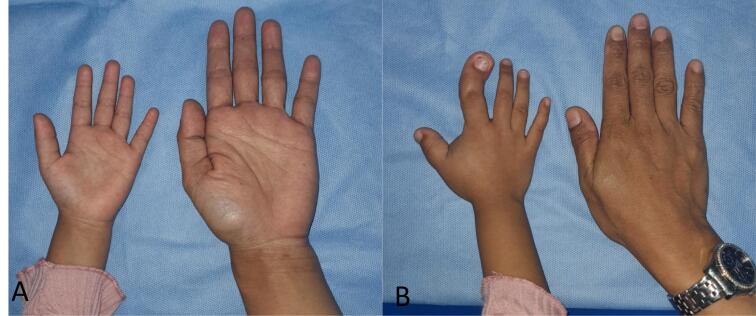
Comparison of the patient's palm with her mother. A. View of the palmar of the right hand. B. View of the dorsum of the right hand.

On examination the first and second fingers of the right hand appear larger than the adjacent fingers, with finger 2 measuring 6 × 1.9 cm and finger 1 measuring 6 × 2 cm. Angulation is present measured at 20 degree, directed towards the ulnar side on the distal dan middle phalanx (Fig. 1) There is discontinuity observed, no tenderness, the sensation is detectable at the base, middle, and tip of the second finger, as well as at the base and tip of the first finger. Range of motion within normal limits, grasping ability present. Sensory within normal limits, comparable to the surrounding and contralateral side. Comparison with patient's mother, size of the patient's mother first finger is 4 × 1,5 cm, and the second finger is 5 × 1,5 cm (Fig. 2). Radiographs indicated widespread edema and heightened soft tissue density in the 1st and 2nd finger of the right hand, specifically on the palm side.

**Fig. 3:**
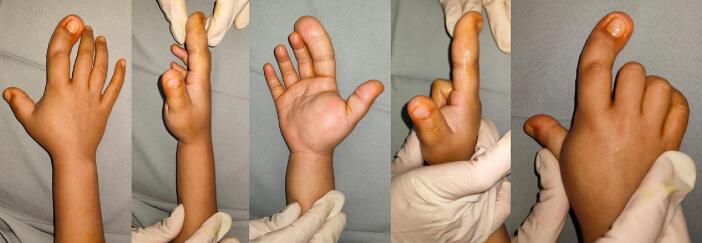
Pre-operation of thumb and index finger right hand.

**Fig. 4:**
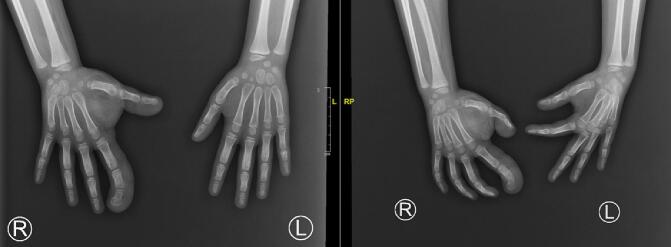
Plain radiographs of both hand before surgery.

Before surgery, we performed the pre-operation examination a week after first admission, the condition is still the same as when it was first examined (Fig. 3). We did a radiologic examination to examine the size of the bone (Fig. 4). In addition to the initial X-ray imaging, further diagnostic evaluations were carried out to confirm the diagnosis of FLH with Macrodactyly and assess its extent. The X-ray images of the patient's right hand revealed an increase in soft tissue density around the affected fingers, indicative of fibroadipose tissue proliferation. Furthermore, the images showed enlargement of the phalanges in the index finger and thumb, consistent with macrodactyly. These findings align with the characteristic radiological features of FLH, including the disproportionate growth of soft tissues and bones in the affected digits. While Magnetic Resonance Imaging (MRI) is recognized as a superior diagnostic tool for assessing soft tissue lesions and was indeed planned for a comprehensive evaluation of the fibrolipomatous hamartoma, the availability of MRI was significantly limited due to long waiting lists. This constraint necessitated prioritizing immediate clinical assessment and intervention based on available diagnostic resources, such as X-ray imaging, which provided initial insights into the condition's impact on bone and soft tissue.

A month after clinical assessment, the surgical intervention for the patient was meticulously planned and executed with the aim of addressing the significant enlargement of the right second finger, while ensuring the preservation of hand function and minimizing potential complications. The procedure comprised several key steps. Debulking: During the surgical debulking of the patient's right hand, specifically targeting the enlarged second finger affected by FLH, careful attention was paid to the composition and location of the tissue being excised. The procedure focused on removing excessive fibroadipose tissue that had proliferated around the median nerve and within the soft tissue matrix of the finger, characteristic of FLH. The excised mass predominantly consisted of a mixture of soft fibrous and adipose tissues. These components are typical of FLH, where fibrous tissue represents the proliferative fibroblasts and collagen deposition around the nerve sheath, and adipose tissue indicates the abnormal fat accumulation within and around the nerve bundles. Notably, the tissue exhibited a soft, pliable consistency, with areas of harder fibrous nodules interspersed within the fatty matrix, reflecting the heterogenous nature of FLH lesions. No significant portions of hard tissue, such as bone, were involved in the debulking process, as FLH primarily affects the soft tissue surrounding the nerve structures. The surgical removal was carefully executed to avoid damage to the nerve itself, ensuring that only the overgrown fibroadipose tissue contributing to the patient's symptoms and functional impairment was excised. This surgical debulking aimed to alleviate the mechanical pressure on the nerve, reduce the bulk of the finger to improve its function and appearance, and, importantly, to obtain tissue for histopathological examination, although, as previously noted, histological analysis was not pursued in this case.

**Fig. 5:**
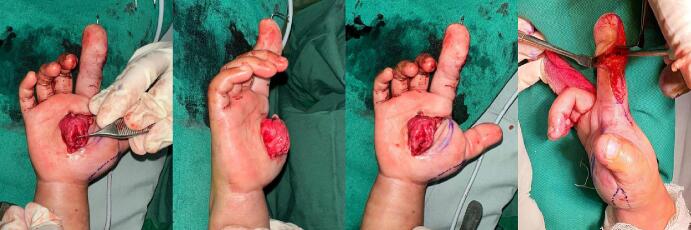
Intraoperation. Debulking, wedge osteotomy dan installation K-Wire digiti 2 middle phalanx right hand.

**Fig. 6:**
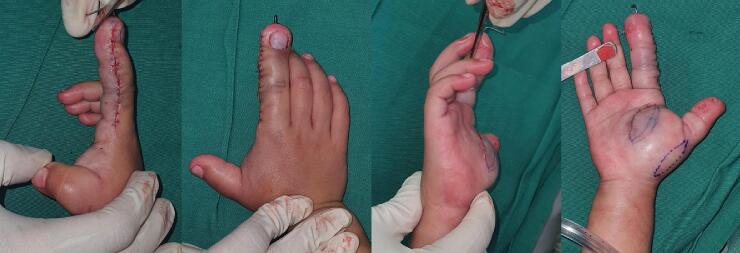
Post operation of the right hand.

Wedge Osteotomy of the Middle Phalanx: To correct the angulation and disproportionate enlargement of the second finger, a wedge osteotomy was performed on the middle phalanx. This involved making precise, angular cuts in the bone to remove a wedge-shaped segment, allowing for the realignment of the finger. The osteotomy was carefully planned based on pre-operative imaging to ensure the correction of angulation while maintaining the finger's length and functionality. K-Wire Insertion: Following the osteotomy, stabilization was achieved by inserting a Kirschner wire (K-Wire) into the middle phalanx. The K-Wire was used to secure the bone in its newly aligned position, ensuring proper healing and consolidation of the osteotomy site. The wire was inserted in a manner that allowed for optimal bone positioning while minimizing interference with finger movement. (Fig. 5). Intraoperative mass was found at palmar region with a suspicion of fibro lipoma, it was decided that the mass was not resected. The patient was scheduled for MRI examination for further work up. Fig. 6, shows the clinical appearance after the surgery.

After the surgical intervention, which included debulking, wedge osteotomy, and K-Wire insertion, the patient underwent a structured post-operative follow-up schedule designed to monitor healing, evaluate the functional recovery of the affected fingers, and mitigate potential complications. The first follow-up, conducted two weeks post-surgery, focused on wound care and the assessment of any immediate post-operative complications. Subsequent follow-ups were scheduled monthly for the first six months, during which the progress of finger function recovery and the effectiveness of the surgical intervention were closely monitored.

The decision to perform surgical intervention on the second finger as a priority was informed by its considerable enlargement, which was more pronounced than other affected areas. This enlargement of the second finger had a significant impact on the patient's hand function, manifesting as impaired gripping ability and dexterity. Addressing the second finger first aimed to alleviate the most immediate functional limitations and discomfort experienced by the patient, with a view to improving quality of life and hand usability in daily activities.

A comprehensive rehabilitation program was initiated early in the post-operative period to enhance the patient's hand functionality and alleviate stiffness. This program, tailored to the patient's specific needs, included physical therapy sessions emphasizing range of motion exercises, strengthening exercises for the hand and fingers, and sensory re-education activities. The patient was also provided with home exercise routines to encourage continuous improvement outside of therapy sessions. Special attention was given to incorporating play-based therapy techniques suitable for the patient's age to maintain engagement and compliance with the rehabilitation process.

Throughout the rehabilitation phase, the patient demonstrated a gradual improvement in grip strength, finger mobility, and a decrease in the stiffness of the right hand. These improvements were significant milestones in the patient's recovery, contributing to an enhanced ability to perform daily activities and an improved quality of life. The patient continues to be monitored regularly to assess long-term outcomes and the potential need for additional interventions.

## 3 Discussion

The wrist is the primary site for the occurrence of the median nerve, constituting 60–70 % of all instances. FLH are rare in the lower extremities, occurring in approximately 17 % of cases. Marek et al. conducted a comprehensive investigation and identified a total of 69 documented incidents of FLH in the plantar foot in a recent study. The lesions were commonly found in the larger, proximal neurons [8,9].

FLH is characterized by the excessive growth of mature fat cells in and around the peripheral nerves. The term “macrodystrophia lipomatosa” is used to describe a condition characterized by excessive bone development and macrodactyly. The initial documentation of this syndrome was provided by Feriz and Barsky. This foundational work laid the groundwork for our current understanding of the condition. Feriz, in 1925, was the first to describe a case of congenital macrodactyly, which he termed ‘macrodystrophia lipomatosa progressiva,’ highlighting the progressive and lipomatous nature of the overgrowth affecting digits. Subsequently, Barsky further elaborated on the condition in 1967, delineating the pathophysiological mechanisms and establishing the clinical characteristics that define macrodystrophia lipomatosa. Their descriptions emphasized the disproportionate growth of soft tissues and bones in the affected limbs, with a particular focus on the fibro-adipose proliferation surrounding nerve bundles, a hallmark of FLH. Overgrowth in the palm, dorsum, and forearm can worsen the situation. The median nerve is the most frequently impacted, with additional affected nerves including the ulnar and radial nerves, among others. Nevertheless, there have been occasional instances of cranial nerve involvement as well [1,2].

The etiology of macrodactyly remains elusive. However, the spread of the condition across the body, especially in areas innervated by the median nerve, and its occurrence in neighboring fingers in cases of multiple-finger macrodactyly, as well as tissue proliferation in the palm, indicate that the disease mechanisms might be significantly influenced by the sensory neurons in those regions. This inference is particularly persuasive for macrodactyly instances that occur near the terminal part of the nerve's pathway. Similarly, other research has noted analogous patterns in progressive macrodactyly cases, where the growth rate of the affected finger decreases following surgical intervention on the implicated nerves [6].

Nerve territory–oriented macrodactyly refers to the main type of authentic macrodactyly, which is characterized by substantial expansion of a peripheral nerve that provides innervation to the affected area. Furthermore, histological examination demonstrates the infiltration of adipose tissue within neural fascicles. Nevertheless, the nerve experienced enlargement due to plexiform schwannomatosis. The NF2 gene is responsible for encoding the protein merlin, which acts as a tumor suppressor. Merlin is involved in regulating pathways of signaling that promote cell division and proliferation, such as the mitogen-activated protein kinase and phosphoinositide-3 kinase cascades. Merlin's atypical conduct can interfere with the local cellular processes that facilitate cell division, while plexiform schwannomatosis has the potential to induce excessive development of the entire finger through unknown neurotrophic mechanisms. [6]

The clinical medical diagnosis of macrodactyly includes a wide range of illnesses, involving both acquired and congenital diseases. These conditions may encompass dactylitis, stroke, Still's disease, osteoid osteoma, lymphangioma, and hemangioma. [1]

FLH are mostly characterized by the abnormal expansion of a finger, hand/ft, or complete limb after birth. These occurrences are commonly noticed during the first three decades of life. One can see a gradual progression of increasing discomfort, tenderness, decreased sensibility, and unusual feelings, accompanied by the development of a mass that puts compression on the nerves. The syndrome of carpal tunnel might be a subsequent manifestation in specific circumstances. The imaging results indicate that the peripheral nerves are larger, infiltrated with lipomatous tissue, and display a coaxial cable-like appearance [1].

FLH are distinguished by the existence of a pliable, gray-yellow, elongated growth that has extensively infiltrated and substituted portions of a significant nerve and its offshoots. The proportions can range from a little, uncomplicated aberrant growth to a significant, intricate disorder known as lipomatosis, which affects all nerves in the upper limbs. The histological examination shows the existence of mature fatty and connective tissue infiltrating the epineural and perineural spaces. The presence of pseudo-onion bulb and metaplastic new bone formation may indicate the involvement of affected nerves [1].

The diagnosis of Neural Fibrolipoma was carefully deduced through a combination of clinical examination findings, radiological evidence, and consideration of the patient's symptomatology. Initially, the physical presentation of the patient's hand, characterized by the disproportionate enlargement of the index finger and thumb, alongside the described pain and sensory alterations, raised the suspicion of a condition affecting both the soft tissue and nerve structures. While the absence of histological confirmation is a limitation in this case, the diagnosis of Neural Fibrolipoma was strongly supported by the clinical presentation, radiological findings, and the characteristic features observed during surgery. Nevertheless, we acknowledge the importance of histology in conclusively diagnosing such conditions and aim to overcome these hurdles in future cases to ensure a comprehensive diagnostic and therapeutic approach.

In the exploration of Fibrolipomatous Hamartoma (FLH) within this report, understanding the possible microscopic variations plays a crucial role in distinguishing FLH from other similar conditions, such as neural tumors and diffuse lipomatosis. For instance, Morton's neuroma showcases degenerative changes, inflammation, and fibrous tissue formation within the nerve, offering a contrast to the presentation of FLH, which is marked by small cutaneous lesions with delicate spindle cells in overlapping fascicles or swirls, frequently alongside adipose tissue. Similarly, diffuse lipomatosis, characterized by widespread growth of mature adipose tissue affecting large areas of limbs or torso without nerve involvement, presents a differential diagnosis. The delineation of these conditions underlines our report's goal: to elucidate the unique histopathological features of FLH, facilitating accurate diagnosis and informing effective treatment strategies. This distinction is pivotal, as it underscores the necessity of precise diagnostic criteria to navigate the complex landscape of conditions presenting with macrodactyly and soft tissue overgrowth, thereby refining our understanding and management of FLH [1].

FLH currently lacks an efficient therapeutic approach. Performing a full removal of the fibrofatty growth is not recommended due to the potential for causing sensory or motor impairments [1].

Hand tumors originating from the peripheral nervous system constitute less than 5 % of all hand tumors. Schwannomas (neurilemmomas) and neurofibromas are the predominant peripheral nerve tumors encountered in the hand. FLH of the nerve is an uncommon benign neoplasm with an uncertain origin. The n. median is commonly impacted, although there have been documented cases of other nerves being affected as well. The occurrence of this disorder in the peripheral level of the fingeral nerves is rare [1,5,10].

A case of multiple FLH in a kid with macrodactyly of both hands has been documented by Gupta A and colleagues. FLH are distinguished by the anomalous hypertrophy of a finger, palm, or sole since birth. Plaza and other researchers recorded 13 cases of fibrolipomatous hamartoma, which is characterized by abnormal growths on one side of the thenar regions, fingers, or both. [1,5,10]

A crucial diagnostic evaluation of macrodactyly is required to rule out any underlying etiologies, such as FLH of a neural. Prompt surgical intervention for this illness has the capacity to prevent long-lasting damage. FLH with macrodactyly is an uncommon disorder defined by an anomalous proliferation that varies in magnitude from a tiny abnormality to a substantial tumor that impacts the entire limb. [1,5,10]

Diagnosing FLH poses a challenge due to its infrequent occurrence and the potential for misdiagnosis or oversight. The diagnostic evaluation should encompass a comprehensive range of potential causes, such as intraneural lipoma, neurilemmoma, neurofibroma, intraneural hemangioma, and Dejerine-Sottas illness. [2]

Radiologically, the X-ray findings of increased soft tissue density and bone enlargement in the affected fingers provided preliminary evidence supporting the presence of a fibro-adipose proliferative process. However, Neural Fibrolipoma, by definition, involves the abnormal proliferation of fibrous and adipose tissue within the nerve sheath, necessitating further evidence to confirm the diagnosis.

The absence of immediate access to Magnetic Resonance Imaging (MRI) due to long waiting times posed a challenge. Nonetheless, the clinical and radiological findings strongly suggested a fibrolipomatous hamartoma. This conclusion was supported by the typical presentation of macrodactyly associated with the condition and the pattern of symptoms reflecting nerve involvement.

Upon eventual MRI examination, which was conducted as soon as available, the diagnosis was conclusively confirmed. The MRI displayed the characteristic ‘coaxial cable’ sign, with images revealing thickened nerve fascicles encased in hyperintense fat, a definitive hallmark of Fibrolipomatous Hamartoma. This imaging evidence, coupled with the clinical presentation and X-ray findings, solidified our diagnosis of Neural Fibrolipoma.

The growth commonly appears within the first decade of life or in the following years. Patients seek medical intervention either because of tumor expansion or due to the development of neuritic problems resulting from the expanding mass. Macrodactyly, a disorder defined by hypertrophy of the nerves in a specific location, can be detected during the physical examination, according to findings by Kelikian et al. This can be observed as a noticeable increase in the size of the affected fingers, along with the presence of a palpable soft tissue mass. [2,3]

Patients may also have paresthesia, or abnormal sensations, as well as varying degrees of sensory impairments. Macrodactyly, also known as nerve territory enlargement, is frequently observed, with prevalence ranging from 62 % to 78 %. This trait is commonly the main indicator that triggers clinical concern for the medical diagnosis. [11,12]

Radiology scanning often shows an increase in the density of soft tissues, along with the presence of exostoses or osteochondromas in close proximity to the site of the abnormality. These findings should also elicit suspicion for fibrous dysplasia of the bone. MRI, an advanced diagnostic modality, enables the viewing of both the nerves and the tumor, thereby offering valuable information. Furthermore, these approaches can unveil the unique visual characteristics of the lesion, commonly characterized as resembling “spaghetti” or “co-axial cable.” [3,7].

The physical examination and scans assist in reducing the spectrum of potential diagnoses. A conclusive diagnosis is achieved through the collection of tissue samples followed by a thorough investigation by a pathologist [2,13].

Due to lack of randomized controlled studies on the treatment of lipomatosis of the nerve, controversy still arises regarding the optimal approach to the problem. Both the surgeon and the patient must weigh the potential risks and benefits of surgery against those of conservative management. The conservative approach in managing FLH includes carpal tunnel decompression, fibro fatty sheath debulking, microsurgical dissection of the neural elements, and observation in asymptomatic patients. The definitive approach includes excision of the involved nerve with or without grafting.

The differential diagnosis for Fibrolipomatous Hamartoma (FLH) encompasses several conditions that manifest with similar clinical and radiological features, such as macrodactyly, soft tissue overgrowth, and nerve involvement. Key conditions to consider include:

1. Neurofibromatosis: Characterized by multiple neurofibromas and café-au-lait spots. Differentiation relies on the presence of systemic manifestations, genetic testing for NF1 and NF2 mutations, and the absence of the characteristic ‘coaxial cable’ MRI appearance seen in FLH.
2. Lipomatosis: Distinguished by diffuse proliferation of adipose tissue without specific nerve involvement. Diagnosis is aided by imaging studies showing extensive adipose tissue growth that lacks the nerve-entrapped appearance typical of FLH.
3. Morton's Neuroma: Presents as a localized painful mass in the foot, often confused with FLH when occurring in the hand. Differentiated through clinical presentation (localized pain, typically between the third and fourth toes) and ultrasound or MRI, which shows a benign growth around the digital nerve in the foot, not associated with the widespread fibroadipose proliferation of FLH.
4. Proteus Syndrome: A rare condition involving asymmetric overgrowth of bones, skin, and other tissues. Differentiation from FLH is based on the broader spectrum of overgrowth and the presence of other anomalies not limited to soft tissue and nerve involvement.

To accurately differentiate FLH from these conditions, a comprehensive approach is employed, incorporating:

1. Clinical Examination: Detailed history and physical examination to assess for systemic involvement and specific signs pointing towards or away from FLH.
2. Imaging Studies: MRI is pivotal in diagnosing FLH, showcasing the ‘coaxial cable’ sign and the fibroadipose proliferation around nerves. Comparison with X-ray and ultrasound findings aids in differentiating from other conditions.
3. Genetic Testing: Essential for conditions like Neurofibromatosis, where specific genetic mutations are diagnostic.
4. Histopathological Examination: While not always necessary for FLH diagnosis, histology can provide definitive differentiation by revealing the unique cellular composition of FLH compared to other proliferative or neoplastic conditions.

## 4 Conclusion

Our case report of a young patient with Fibrolipomatous Hamartoma (FLH) and associated macrodactyly emphasizes the condition's distinctive clinical and radiological features. Surgical intervention, particularly debulking and corrective osteotomy, played a critical role in managing the patient's symptoms and improving hand function. This report underscores the importance of a multidisciplinary approach to accurately diagnose and effectively treat rare conditions like FLH, contributing to better patient outcomes. Future research should continue to explore optimal diagnostic and treatment strategies for FLH and its complications.

## Ethical approval

Ethical approval for this study (Ethical Committee N° NAC 207) was provided by the Ethical Committee of Dr. Hasan Sadikin General Hospital, Bandung, Indonesia on 15 November 2023.

## Guarantor

Hardisiswo Soedjana.

Betha Egih Riestiano.

Lisa Y. Hasibuan.

Valdi Muharam Kusumadiningrat.

## Declaration of competing interest

The authors declare no conflicts of interest related to this study.
